# Doping palladium with tellurium for the highly selective electrocatalytic reduction of aqueous CO_2_ to CO†
†Electronic supplementary information (ESI) available: Experimental details, TEM and HRTEM images, XPS survey, linear sweep voltammetry, cyclic voltammograms, faradaic efficiency, and DFT calculation details. See DOI: 10.1039/c7sc03018e


**DOI:** 10.1039/c7sc03018e

**Published:** 2017-11-06

**Authors:** Hengcong Tao, Xiaofu Sun, Seoin Back, Zishan Han, Qinggong Zhu, Alex W. Robertson, Tao Ma, Qun Fan, Buxing Han, Yousung Jung, Zhenyu Sun

**Affiliations:** a State Key Laboratory of Organic-Inorganic Composites , Beijing University of Chemical Technology , Beijing 100029 , China . Email: sunzy@mail.buct.edu.cn; b Beijing National Laboratory for Molecular Sciences , Key Laboratory of Colloid and Interface and Thermodynamics , Institute of Chemistry , Chinese Academy of Sciences , Beijing 100190 , China . Email: hanbx@iccas.ac.cn; c Graduate School of EEWS , Korea Advanced Institute of Science and Technology (KAIST) , Daejeon 34141 , Republic of Korea . Email: ysjn@kaist.ac.kr; d Department of Materials , University of Oxford , Oxford , OX1 3PH , UK

## Abstract

Designing highly selective and energy-efficient electrocatalysts to minimize the competitive hydrogen evolution reaction in the electrochemical reduction of aqueous CO_2_ remains a challenge. In this study, we report that doping Pd with a small amount of Te could selectively convert CO_2_ to CO with a low overpotential. The PdTe/few-layer graphene (FLG) catalyst with a Pd/Te molar ratio of 1 : 0.05 displayed a maximum CO faradaic efficiency of about 90% at –0.8 V (*vs.* a reversible hydrogen electrode, RHE), CO partial current density of 4.4 mA cm^–2^, and CO formation turnover frequency of 0.14 s^–1^ at –1.0 V (*vs.* a RHE), which were 3.7-, 4.3-, and 10-fold higher than those of a Pd/FLG catalyst, respectively. Density functional calculations showed that Te adatoms preferentially bind at the terrace sites of Pd, thereby suppressing undesired hydrogen evolution, whereas CO_2_ adsorption and activation occurred on the high index sites of Pd to produce CO.

## Abstract 2

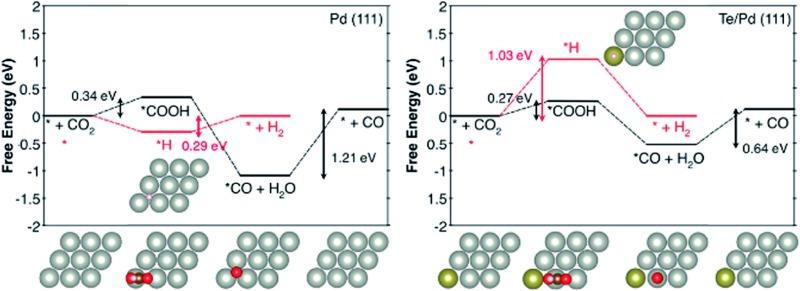
The doping of Pd with a small amount of Te can selectively convert CO_2_ to CO with a low overpotential.

## Introduction

Steady increases in the atmospheric levels of CO_2_ and increasing energy demands have intensified concerns about the adverse CO_2_ effects on climate change and energy supplies. The electrochemical reduction of CO_2_ to value-added fuels and chemicals using renewable energy resources (solar, tidal, and wind) provides a “clean” and efficient way to mitigate energy shortage and lower the global carbon footprint.^1^ Nonetheless, the linear molecule CO_2_ is stable and chemically inert with a low electron affinity and large energy gap (13.7 eV) between its lowest unoccupied molecular orbital (LUMO) and highest occupied molecular orbital (HOMO). CO_2_ transformation is dominated by nucleophilic attacks at the carbon, and is an uphill process requiring a substantial input of energy (∼750 kJ mol^–1^ is required for the dissociation of a C=O bond). A variety of metallic electrodes have been developed to accelerate such kinetically slow reduction reactions, and the product distribution is strongly dependent on the nature of the electrode surface. Despite recent advances achieved in the electrocatalytic reduction of CO_2_, this field still faces the challenges of (1) large overpotential (or low energetic efficiency); (2) slow electron transfer kinetics, resulting in low exchange current densities; (3) unsatisfactory selectivity, implying a costly separation step.^2^–^4^ In addition, proton reduction to evolve H_2_ usually occurs as a severe competitive reaction, especially in aqueous electrolytes, affecting the CO_2_ reduction selectivity and efficiency.

The electrochemical reduction of CO_2_ to CO is a simple two-electron transfer process. By transferring a concerted proton-electron (H^+^/e^–^) from solution to the adsorbed species, a CO_2_ molecule is reduced to a carboxyl intermediate, *COOH. Another possible route to generate *COOH is through a decoupled electron and proton transfer, involving the formation of a CO_2_˙^–^ radical that is adsorbed at the electrode surface. A second H^+^/e^–^ can subsequently attack the oxygen atom (OH) in *COOH to form H_2_O (l) and CO.^5^ Metals such as Au, Ag, Zn and Pd bind *COOH tightly enough for further reduction to yield the *CO intermediate. The *CO intermediate is weakly bound to the electrode surface, and CO desorbs from the electrode as a major product.^6^,^7^ Recently, Bao *et al.* demonstrated the size effect of Pd particles on the electrocatalytic reduction of CO_2_. The faradaic efficiency for CO production over 10.3 nm nanoparticles (NPs) was reported to be only 5.8% at –0.89 V (*vs.* a reversible hydrogen electrode, RHE).^2^ In addition to control of the size, tuning the surface strain of Pd^8^ and alloying with a second metal (Pd–Au,^9^ Pd–Ni,^10^ Pd–Cu,^11^,^12^ and Pd–Pt^13^,^14^) can enhance the energy efficiency and CO selectivity for CO_2_ reduction. Density functional theory (DFT) calculations have revealed that the low coordinate sites (corners/edges) of the Pd particles facilitate CO_2_ adsorption and the formation of *COOH, compared with the terrace sites.^2^ From this scenario, the addition of foreign metal adatoms to tailor the electronic and geometric properties of the Pd nanocrystals can substantially modify their corresponding catalytic activities. However, there have been limited studies on CO_2_ reduction over metal NPs regarding the impact of doping effects, and it is of great interest to gain further understanding about the doping effects at the atomic level.

We report herein that doping Pd nanocrystals with Te significantly enhances the electrochemical reduction of aqueous CO_2_ to CO, affording a much higher CO faradaic efficiency (FE), CO partial current density, mass activity, and formation turnover frequency with respect to the undoped Pd catalyst. DFT calculations reveal that Te adatoms preferentially bind at the terrace sites of Pd, which can suppress unwanted hydrogen evolution in aqueous electrolysis.

## Results and discussion

**Fig. 1:**
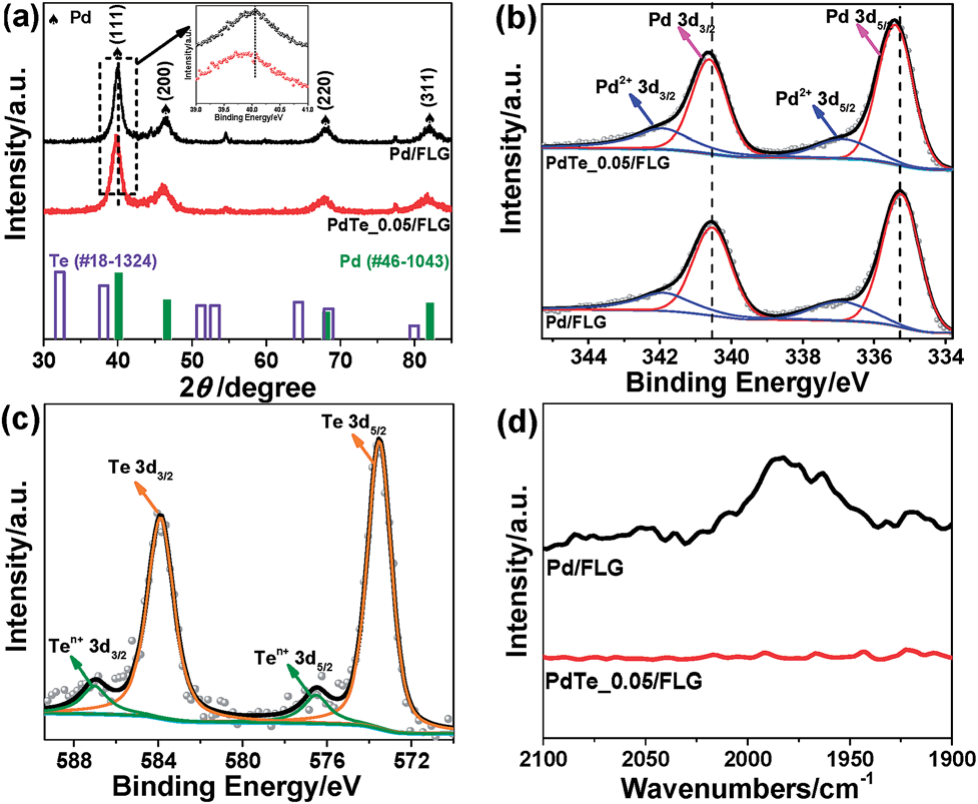
(a) The XRD patterns, (b) Pd 3d and (c) Te 3d XPS spectra, and (d) the CO FTIR spectra of 30 wt% Pd/FLG (top) and 30 wt% PdTe_0.05/FLG (bottom). The bottom panel in (a) shows the reference patterns for Te (hollow column, #18-1324) and metallic Pd (solid column, #46-1043).

Pd NPs doped with Te were deposited on the surface of few-layer graphene (FLG) using the ultrasonication-facilitated reduction of the PdCl_2_ and TeCl_4_ precursor mixture. No capping ligands were used during the process, resulting in clean surfaces for the NPs and avoiding the problem that the stabilizer molecules adversely affect the particle properties. The X-ray diffraction (XRD) patterns of Pd/FLG and PdTe/FLG are given in Fig. 1a, S1 and S2 (ESI†). All of them showed four pronounced diffraction peaks at approximately 40.1°, 46.7°, 68.1°, and 81.8°, corresponding well to the (111), (200), (220) and (311) planes of face-centered cubic (fcc) Pd (PDF#46-1043), respectively. The (111) reflection is shifted toward lower 2*θ* after Te doping (inset of Fig. 1a and S1b, ESI†), which may be caused by the lattice mismatch upon surface decoration or by the presence of interstitial heteroatoms (H or Te).^15^,^16^ The average crystallite sizes were estimated to be about 8.3 and 11.0 nm for 30 wt% Pd/FLG and 30 wt% PdTe_0.05/FLG, respectively, from the (111) reflection utilizing Scherrer’s equation relating the coherently scattering domains with the Bragg peak widths: *L* = *kλ*/*B* cos(*θ*), in which *k* = 0.89 for spherical particles and *B* is the full angular width at half-maximum of the peak in radians.^17^ We noted that the particle size increased upon the introduction of Te into Pd, and reaches about 14.7 nm at a Te/Pd atomic ratio of 0.15 (Fig. S3, ESI†). Additionally, improving the loading content of PdTe led to an increase in the particle sizes (Fig. S3, ESI†).

X-ray photoelectron spectroscopy (XPS) was employed to provide insight into the surface composition of the resulting PdTe/FLG (Fig. 1b, c and S4, ESI†). The Pd 3d XPS spectrum of PdTe/FLG (top panel in Fig. 1b) consists of asymmetric Pd 3d_5/2_ and 3d_3/2_ peaks centered at 335.3 and 340.6 eV, respectively, characteristic of Pd^0^.^18^ The Pd 3d binding energies (BEs) of PdTe/FLG are shifted to higher values compared with the BEs of Pd/FLG (the bottom panel in Fig. 1b), suggesting electron transfer from Pd to Te.^19^ This implies that the Pd d-band center shifts down when Pd is doped with Te, according to the d-bond theory.^20^–^22^ The Te 3d XPS spectrum (Fig. 1c) exhibited peaks at 573.8 eV (Te 3d_5/2_) and 583.5 eV (Te 3d_3/2_), indicative of Te^0^. The other weaker doublets at higher BEs, displayed in Fig. 1b and c, are associated with Pd^2+^ and Te^4+^, respectively, which may result from the slight oxidation of the corresponding metal in contact with air.^17^,^23^

Probing the CO adsorbed on the Pd nanoparticle surfaces by Fourier transform infrared (FTIR) spectroscopy allows one to examine the Pd surface features modified by the Te adatoms. In Pd/FLG, CO was observed to be bonded on the (111) terrace Pd^0^ sites at ∼1970 cm^–1^ in a bridging mode (top panel in Fig. 1d).^24^,^25^ However, there is no discernible bridging CO peak at a 1 : 0.05 mole ratio of Pd to Te (bottom panel in Fig. 1b), indicating full coverage of possibly most of the terrace sites at such a low Te doping level to block the active sites for the hydrogen evolution reaction.^15^

**Fig. 2:**
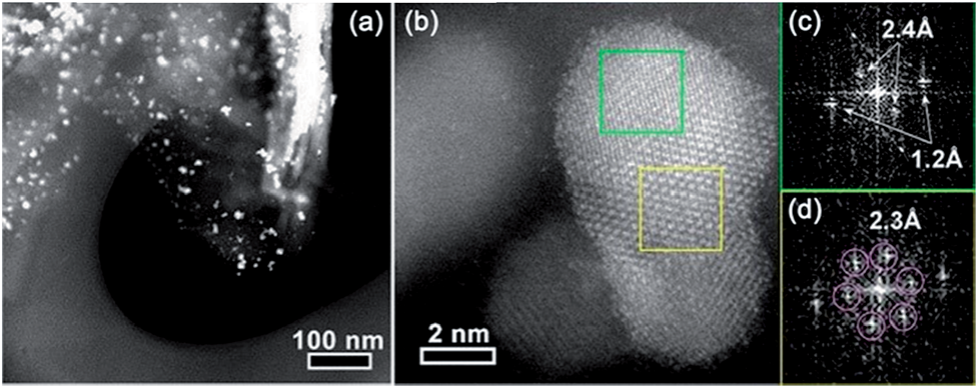
(a) The low- and (b) high-magnification STEM images of 30 wt% PdTe_0.05/FLG. The bright white streak on the right side of the FLG shown in image a is likely due to part of the FLG being folded up. (c) and (d) Fast Fourier transforms (FFTs) of the regions marked by the green and yellow squares in (b), respectively.

The typical scanning electron microscopy (SEM) images showed a large number of graphitic flakes with lateral sizes of 300 nm to 5 μm lying flat on top of each other (Fig. S5a, S6a and b, ESI†). No free-standing particles detached from FLG and no large particle agglomerates were observed. The formation of highly dispersed PdTe NPs was confirmed by *in situ* energy-dispersive X-ray spectroscopy (EDX) (Fig. S2b, ESI†) together with elemental mapping (Fig. S6c–e, ESI†). High-angle annular dark field scanning TEM (HAADF-STEM) observation showed that isolated crystalline NPs were evenly distributed on the surface of the FLG (Fig. 2a and S7d, ESI†). The average particle size of the PdTe NPs is about 9.7 nm, slightly bigger than that of Pd NPs (∼8.6 nm), agreeing reasonably with the XRD results (Fig. S9, ESI†). Clearly, there are two distinctive types of lattice observed in an individual crystal of Te doped Pd (Fig. 2b). EDX confirms both Pd and Te signals (Fig. S7a–c, ESI†). The centered crystal with a smaller lattice is covered by an outer surface lattice with larger spacing (Fig. 2c and d). The inner lattice agrees well with the (311) spacing of fcc Pd while the outer lattice with a d-spacing of about 0.24 nm corresponds to hexagonal close-packing (hcp) Te.^15^

**Fig. 3:**
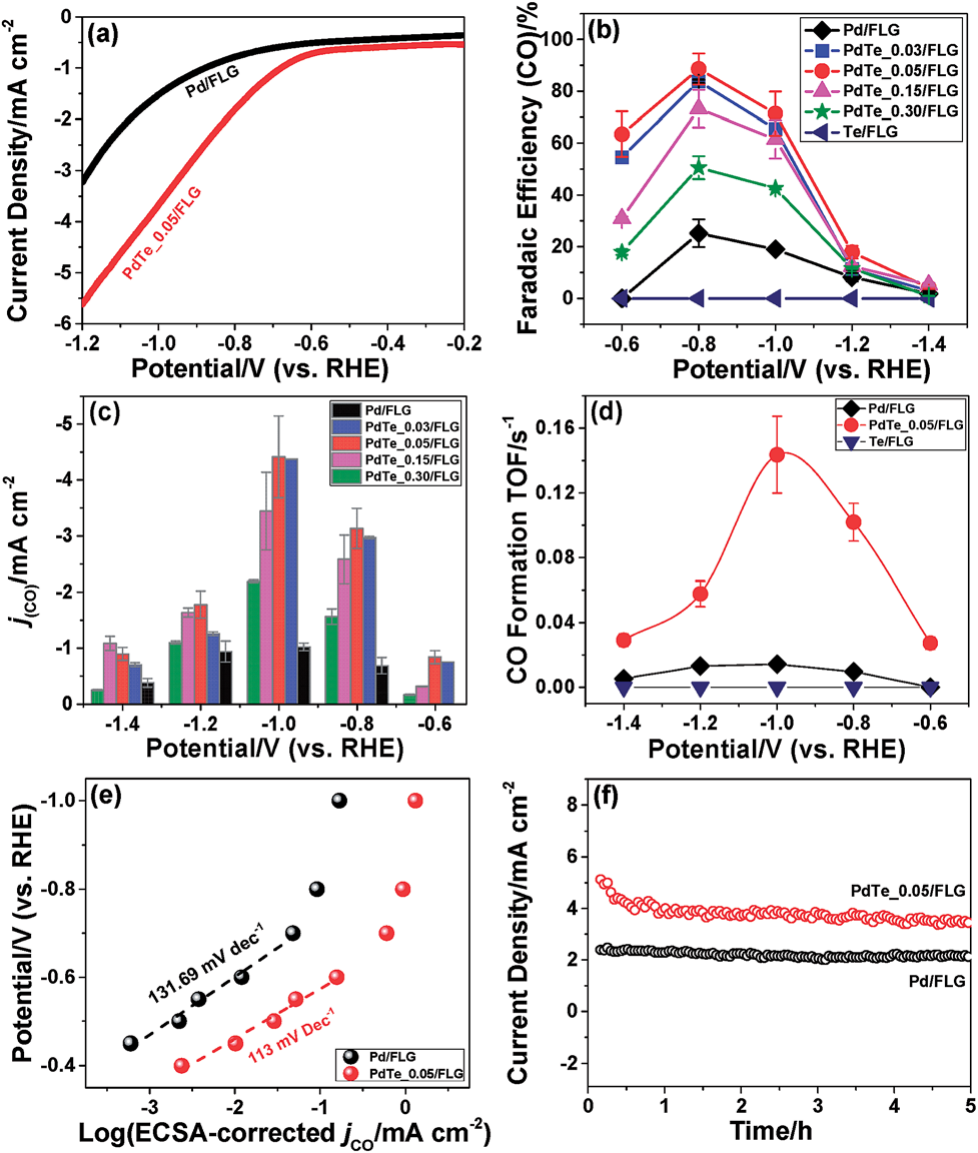
(a) The LSV results of the 30 wt% Pd/FLG and 30 wt% PdTe_0.05/FLG electrodes in CO_2_-saturated 0.1 M KHCO_3_ with a scan rate of 5 mV s^–1^. (b) The faradaic efficiency of CO and (c) partial current density of CO formation dependent on the Te doping ratios at various applied potentials. (d) The CO formation TOF of 30 wt% Pd/FLG, 30 wt% PdTe_0.05/FLG, and 30 wt% Te/FLG at different applied potentials. (e) The ECSA-corrected Tafel plots for CO production at applied potentials and (f) the long-term durability test at –0.8 V (*vs.* a RHE) over 30 wt% Pd/FLG and 30 wt% PdTe_0.05/FLG electrodes.

The CO_2_ reduction activities of all the catalysts were tested in a typical three-electrode electrochemical system. Fig. S10a and b† show the linear sweep voltammetry (LSV) results of 30 wt% Pd/FLG and 30 wt% PdTe_0.05/FLG in 0.1 M aqueous KHCO_3_ solution saturated with argon or CO_2_, respectively. It can be seen that the Te-doped sample (30 wt% PdTe_0.05/FLG) exhibited a more positive onset potential and a current density nearly twice as high as that of 30 wt% Pd/FLG at –1.2 V (*vs.* a RHE) (Fig. 3a), indicating that the addition of Te atoms enhanced the CO_2_ reduction reaction. Controlled potential electrolysis of CO_2_ was further performed at potentials between –0.6 and –1.4 V (*vs.* a RHE) in a CO_2_-saturated 0.1 M KHCO_3_ solution (pH 6.8) at room temperature under atmospheric pressure. Under these reaction conditions, only CO and H_2_ were detected by gas chromatography (GC).

The effects of the Te doping content on the FE for the formation of CO at –0.6 to –1.4 V (*vs.* a RHE) are shown in Fig. 3b. At –0.6 V (*vs.* a RHE), 30 wt% Pd/FLG generated only H_2_ (Fig. S11a, ESI†), whereas the Te-doped Pd catalysts produced a mixture of CO and H_2_ with CO selectivity of up to ≈64% for the 30 wt% PdTe_0.05/FLG. The CO FE increased with decreasing Te doping content at different applied potentials, reaching a maximum at a Pd/Te molar ratio of 1 : 0.05. Further reduction in the Te doping content led to a slight decrease of the CO FE. Compared with the Pd wire electrode exhibiting a CO FE of less than 6%,^26^ the catalysts with Pd/Te molar ratios of 1 : 0.03 and 1 : 0.05 both showed a maximum CO FE exceeding 80% at –0.8 V (*vs.* a RHE, overpotential of 690 mV). In particular, the CO FE for that for 30 wt% PdTe_0.05/FLG approached ≈90%, 3.7-fold higher than 30 wt% Pd/FLG. This catalyst also significantly outperforms Pd NPs of a similar size (≈10.3 nm) which exhibit a CO FE of only 5.8% at –0.89 V (*vs.* a RHE, overpotential of 780 mV) as reported in literature.^2^ Likewise, the Te-doped Pd catalysts displayed larger CO partial current densities at various reduction potentials (Fig. 3c). The mass activities of the catalysts at the applied potentials followed a similar trend with their CO partial current densities, further confirming the better performance of these Te-doped catalysts for CO_2_ reduction (Fig. S11b, ESI†). We also found that the catalyst with a 30 wt% PdTe loading gave the highest CO FE at the applied potentials among Te-doped catalysts with a constant Pd/Te molar ratio of 1 : 0.05 (Fig. S12a, ESI†).

The CO formation turnover frequency (TOF) (Fig. 3d), a measure of the pre-site activity of catalysts to produce CO, was 0.14 s^–1^ for the 30 wt% PdTe_0.05/FLG *versus* 0.014 s^–1^ for 30 wt% Pd/FLG at –1.0 V (*vs.* a RHE, overpotential of 890 mV). However, the CO formation TOF was zero for 30 wt% Te/FLG at all applied potentials, indicating that Te could not produce CO under the aqueous electrolysis of CO_2_.

The electrocatalytically active surface area (ECSA)-corrected Tafel slope, an indication of the kinetics for CO formation,^27^ was 113 mV dec^–1^ for 30 wt% PdTe_0.05/FLG, closer to the value of 118 mV dec^–1^ expected for the rate-determining step at an electrode compared to a Tafel slope of 131.69 mV dec^–1^ for 30 wt% Pd/FLG (Fig. 3e). This indicates that the formation of an adsorbed HCOO* intermediate on the catalyst surface determines the reaction rate in both cases, but the Te-doped catalyst has better kinetics for CO_2_ reduction.^28^

The long-term performances of 30 wt% PdTe_0.05/FLG and 30 wt% Pd/FLG were evaluated at a constant potential of –0.8 V (*vs.* a RHE) for 5 h (Fig. 3f), and the current density retained 3.5 mA cm^–2^ and 2 mA cm^–2^, respectively.

The effect of Te doping on the activity and selectivity for the electrochemical reduction of CO_2_ was investigated using DFT calculations. We first compared the thermodynamic preference of Te doping on various Pd surfaces (Fig. S14†), and found that Te doping is most favourable at the Pd (111) surface, which is in agreement with the XRD observation (Fig. 1a). Thus, it is reasonable to assume that Te prefers to replace a Pd atom in the (111) surface, and this affects the catalytic properties of this surface. On the basis of this result, we modelled the Pd (111) surface with and without Te decoration. Considering that a favourable adsorption of H leads to H-covered Pd surfaces under the CO_2_ reduction condition,^29^ we also modelled the Pd (111) surface covered with 1 monolayer of *H and the Te/Pd (111) surface covered with 2/3 monolayers of *H, denoted as 1 ML *H–Pd (111) and 2/3 ML *H–Te/Pd (111), respectively (Fig. S15†).

**Fig. 4:**
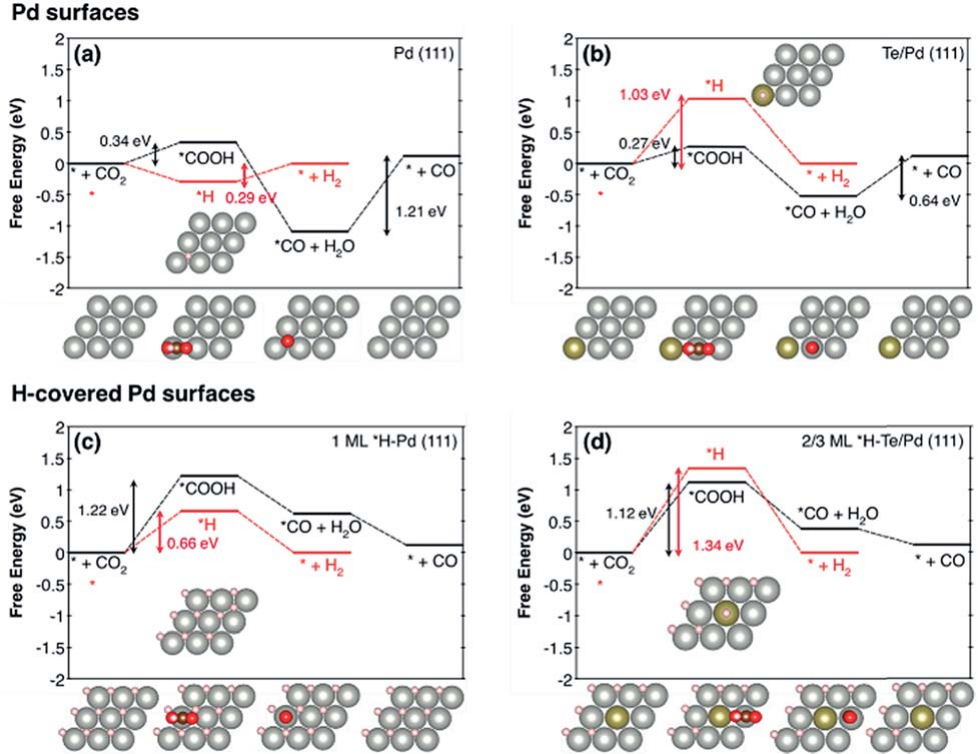
The free energy diagrams of CO_2_ reduction to CO (black) and the H_2_ evolution reaction (red) for (a) Pd (111), (b) Te/Pd (111), (c) 1 ML *H–Pd (111), and (d) 2/3 ML *H–Te/Pd (111). Top views of the first layer of optimized geometries are shown. The light grey, dark grey, gold, red, and white balls indicate Pd, C, Te, O, and H, respectively. We note that * represents a surface site for adsorption.

Comparing the free energy diagrams of the desired CO_2_ reduction and unwanted H_2_ evolution reaction with and without Te decoration, we found three noteworthy points, in good agreement with the experimental measurement (Fig. 4). First, when Te is introduced to the surface layer, the energy required to form *COOH decreased by 0.07 eV and 0.10 eV for the Pd and H-covered Pd surfaces, respectively. This result can be linked to the substantially increased experimental partial current density of CO formation of PdTe/FLG compared to Pd/FLG (Fig. 3c). Second, the Te dopant facilitated the desorption of produced CO (0.64 eV), whereas this desorption step is problematic with the higher desorption energy (1.21 eV) on the Pd surface. We expect that the weakening of the *CO binding on Te/Pd (111) is responsible for the absence of the CO peak in the FTIR spectra (Fig. 1d). Third, and most importantly, Te doping significantly destabilized the *H binding energies on the Te top site for both the Pd and H-covered Pd surfaces by 0.74 eV and 0.68 eV, respectively. We note that the geometry optimization of H adsorption with the usual three-fold hollow site (Te–Pd–Pd) as an initial geometry leads to the H atom migrating from the initial hollow site to the Pd–Pd bridge site (Fig. S15†), implying the tendency of *H to avoid Te atoms. Overall, the Te doped environments tend to repel *H adsorption and yield slightly improved CO_2_ electrocatalysis.

With the help of density of states analysis for the Pd atom with and without Te dopant, we found that the electronic effect of the Te atom on a nearby Pd atom is actually not significant (Fig. S16†), but the main effect of Te is rather to reduce the number of three-fold hollow sites for strong *H adsorption. We further note that the intrinsic improvement of CO_2_ reduction itself, due to Te, would not be that substantial compared to the deterioration of H_2_ evolution, since the low coordinated stepped sites, *i.e.* the (211) surface, are energetically more active for CO_2_ electrocatalysis than the terrace sites, and thus responsible for the catalytic activity.^30^

## Conclusions

In summary, we demonstrate that doping Pd with Te permits a significant enhancement in the electroreduction of aqueous CO_2_ to CO. Te-doped Pd NPs with a Pd/Te molar ratio of 1 : 0.05 exhibit a CO faradic efficiency of ∼90% at –0.8 V (*vs.* RHE), which is 3.7-fold higher than the maximum FE of undoped Pd NPs of similar size (∼24%). Such doping also allows 4.3- and 10-fold improvement in the CO partial current density and CO formation turnover frequency. DFT calculations show that Te doping reduces the energy required to form *COOH, weakens *CO binding, and destabilizes the *H binding energies for both the Pd and H-covered Pd surfaces. Importantly, the Te adatoms preferentially bind at the terrace sites of Pd, which can suppress unwanted hydrogen evolution in aqueous electrolysis, whereas CO_2_ adsorption and activation occur on the high index sites of Pd to produce CO.

## Conflicts of interest

The authors of this manuscript have no conflicts of interest.

## Supplementary Material

Supplementary information
