# Supplementary material for: Doping palladium with tellurium for the highly selective electrocatalytic reduction of aqueous CO2 to CO
Source: Chem Sci. 2017 Nov 6;9(2):483–7. doi: 10.1039/c7sc03018e (PMC5868300; doi:10.1039/c7sc03018e)
Supplement: Supplementary file 1 [file SC-009-C7SC03018E-s001.pdf]

Electronic Supplementary Material (ESI) for Chemical Science.
This journal is © The Royal Society of Chemistry 2017

Supporting Information

Doping Palladium with Tellurium for Highly Selective Electrocatalytic Reduction of Aqueous CO_2 to CO

Hengcong Tao,[†a] Xiaofu Sun,[†b] Seoin Back,[c] Zishan Han,[a] Qinggong Zhu,[b] Alex W. Robertson,[d] Tao Ma,[a] Qun Fan,[a] Buxing Han,[*b] Yousung Jung,[*c] and Zhenyu Sun,[*a]

[a]*State Key Laboratory of Organic-Inorganic Composites, Beijing University of Chemical Technology, Beijing 100029, China. E-mail: sunzy@mail.buct.edu.cn*
[b]*Beijing National Laboratory for Molecular Sciences, Key Laboratory of Colloid and Interface and Thermodynamics, Institute of Chemistry, Chinese Academy of Sciences, Beijing 100190, China. E-mail: hanbx@iccas.ac.cn*
[c]*Graduate School of EEWS, Korea Advanced Institute of Science and Technology (KAIST), Daejeon 34141, Republic of Korea. E-mail: ysjn@kaist.ac.kr*
[d]*Department of Materials, University of Oxford, Oxford, OX1 3PH, United Kingdom*
† These authors contributed equally to this work.

Experimental Procedures

Synthesis of few layer graphene (FLG). Pristine layered graphite flakes were used as starting material for graphene exfoliation. Graphene dispersions were prepared by adding graphite to NMP (300 mL) in 500 mL round-bottomed flasks with the aid of bath ultrasonication, a technique commonly applied to accelerate exfoliation. After 12 h of ultrasonication, the dispersions were centrifuged at 3000 rpm for 30 min; the top two-thirds of the dispersions were gently gathered by pipetting. Few-layer graphene powder was obtained by vacuum filtration of the centrifuged dispersions onto porous nylon membranes (Whatman, 0.2 μm pore size, 47 mm membrane diameter) followed by drying at 45 °C overnight.

Synthesis of PdTe_x/ FLG. Typically, 2 mg of graphene was initially dispersed in 10 mL of one precursor or two mixed precursor isopropanol aqueous solution at a designated concentration to form a uniform suspension with the aid of bath sonication for 30 min under N_2 atmosphere. As 1 mL of a $NaBH_4$ isopropanol aqueous solution

(its concentration being 5 times higher than that of the precursor) was added drop-wise into the dispersion containing metal precursor (1 mg mL^{-1} $PdCl_2$ was dissolved in 0.1 M HCl solution) under bath sonication within 2 min, Pd or Te-doped Pd particles were deposited onto graphene. Subsequently, the obtained system in each case was ultracentrifuged and the collected precipitate was washed repeatedly with absolute ethanol and distilled water, and then dried at 45 °C for 12 h.

Characterization. XPS experiments were carried out using Thermo Scientific ESCALAB 250Xi instrument. The instrument was equipped with an electron flood and scanning ion gun. All spectra were calibrated to the C1s binding energy at 284.8 eV. X-ray powder diffraction (XRD) was performed with a D/MAX−RC diffractometer operated at 30 kV and 100 mA with Cu Kα radiation. Scanning electron microscopy (SEM) was carried out using a field emission microscope (FEI Quanta 600 FEG) operated at 20 kV and equipped with an energy-dispersive X-ray spectrometer (EDX). High-angle annular dark field scanning TEM (HAADF-STEM) was conducted using a JEOL ARM200 microscope with 200 kV accelerating voltage. STEM samples were prepared by depositing a droplet of suspension onto a Cu grid coated with a lacey carbon film. Infrared data was collected using a Nicolet 6700 ATR-IR spectrometer with liquid nitrogen-cooled MCT detector. The spectra were obtained by averaging 128 scans with a resolution of 4 cm^{-1} over wavenumbers ranging from 650 to 4000 cm^{-1}.

Electrochemical measurements. For electrochemical reduction of CO_2, the cyclic voltammetry (CV) and linear scan voltammogram (LSV) were performed using rotating disk electrode (RDE) by submersing working electrode in a three-electrode cell. Controlled potential electrolysis of CO_2 was tested in an H-cell system, which was separated by Nafion 117 membrane. Toray carbon fiber paper with a size of 1 cm × 1 cm was used as working electrode. Pt wire and Ag/AgCl electrodes were used as counter electrode and reference electrode, respectively. The potentials were controlled by an electrochemical working station (CHI 760E, Shanghai CH Instruments Co.,

China). All potentials in this study were measured against the Ag/AgCl reference electrode and converted to the RHE reference scale by

E (vs. RHE) = E (vs. Ag/AgCl) + 0.21 V + 0.0591 × pH (Eq. S1)

Electrocatalytic reduction of CO_2 was conducted in CO_2-saturated 0.1 M $KHCO_3$ solution at room temperature under atmospheric pressure. CO_2 was purged into the $KHCO_3$ solution for at least 30 min to remove residual air in the reservoir, then controlled potential electrolysis was performed at each potential for 300 min.

Electrocatalytically active surface area (ECSA) and ECSA corrected-Tafel slope measurements. For Pd, the charge of hydrogen underpotential deposition cannot be precisely aquired due to the interference of hydrogen absorption in Pd. As such, the values of ECSA were estimated on the basis of the reduction charge of surface $Pd(OH)_2$, as reported in previous literature (Fig. S13).[1] A potential of +1.2 V was chosen as the appropriate potential limit because this potential is the upper limit for the formation of $Pd(OH)_2$ in a solution of pH = 1.[1] The ECSA value can be determined by

$$\mathrm{ECSA} = \mathrm{GSA} * (Q_{\mathrm{R}} / Q_{\mathrm{F}}) \qquad \text{(Eq. S2)}$$

where GSA is the the geometric surface area, Q_R is the charge density for reduction of surface $Pd(OH)_2$ on working electrode and Q_F is the charge density for the formation of a fully covered $Pd(OH)_2$ layer. The value of Q_F employed here is 430 μC cm^{-2}, the minimum of the reported values for typical single-crystal Pd surfaces.[2] ECSA-corrected Tafel slopes for CO production (that is, $j_{total} \times \eta_{CO}$/ECSA) were calculated from corresponding ECSA-corrected current densities for CO based on the linear sweep voltammetry curves and the CO Faradaic efficiency.

Number of active sites and turn over frequency (TOF) measurements. To further characterize the catalytic activities of Pd/FLG, PdTe/FLG, and Te/FLG, we applied a roughness factor technique to determine the number of active edge sites of the catalysts. Roughness factor (R_f) was estimated from the ratio of double-layer capacitance (C_{dl}) between the working electrode and its corresponding smooth Pd electrode (assuming that the average double-layer capacitance of a smooth Pd electrode is 20 μF cm^{-2}),[3] R_f

= C_{dl}/20 µF cm^{-2} (Eq. S3). The C_{dl} was determined by measuring the capacitive current associated with double-layer charging from the scan-rate dependence of cyclic voltammetric stripping (Figs. S6d and S9d). A series of CV experiments at different scan rates e.g., 20, 50, 80, 120, 160 and 200 mV s^{-1} were performed in 0.5 M H_2SO_4 to calculate the C_{dl} of each catalyst. All experiments were performed using the same surface area.

The calculated number of active sites for each catalyst was obtained using the equation:

Density of active sites for catalyst (sites/cm^2) = Density of active sites for standard sample (sites/cm^2) × R_f (Eq. S4)

Additionally, the CO formation turn over frequency (TOF) of active sites for CO_2 reduction reaction in the PdTe_x/FLG was calculated at different overpotentials using Eq. S4:

CO formation TOF (s^{-1}) = i_0 (A cm^{-2}) × CO formation FE /([active sites density (sites/cm^2)] × [1.602 ×10^{-19} (C/e$^-$)] × [2e$^-$/CO_2]) (Eq. S5)

Faradaic efficiency (FE) measurement. The FE values of catalysts were calculated using $E_{Faradaic} = \alpha nF/Q$, where α is the number of electrons transferred (α = 2 for CO and H_2 production), n the number of moles for a given product, F Faraday's constant (96,485 C mol^{-1}), Q all the charge passed throughout the electrolysis process (measured by calculating the curve area of current density vs. time plot). CO and H_2 mole fractions of injected samples were calculated using GC calibration curve.

Product analysis of CO_2 reduction electrolysis experiments. The gas-phase product analysis for the electrochemical experiments was carried out using an Agilent 7890B GC system. Two thermal conductivity detectors and flame ionization detector were used to analyze and differentiate injected samples. To characterize the gas product, 20 mL of produced gas in the dead volume of a gas bag (~ 2 L) was injected into the GC at identical experimental conditions e.g., pressure, temperature, and time using sample lock syringe. CO and H_2 peaks were detected at 11.4 and 3.7 min, respectively. The

liquid product was analyzed in DMSO-d_6 with tetramethylsilane (TMS) as an internal standard by ^{1}H nuclear magnetic resonance (NMR) (Bruker Avance III 400 HD spectrometer). No liquid products including formate were detectable by ^{1}H NMR at -0.8 V (vs. RHE) in CO_2-saturated 0.1 M $KHCO_3$ solution. This indicates that CO and H_2 were major products as detected by GC.

Computational Details. Electronic calculations were performed using Vienna Ab initio Simulation Package (VASP).[4] GGA-RPBE[5,6] functional of density functional theory (DFT) was used and projector augment-wave (PAW) method[7,8] was chosen to represent the core-valence interactions. A cutoff energy for pseudopotential was set to 400 eV. Using the calculation setup, a unit cell of face centered cubic Pd was relaxed with (8 × 8 × 8) Monkhorst-Pack mesh, and the lattice parameter was calculated to be 3.97 Å. On the basis of the lattice parameter, (100), (110), (111) and (211) surfaces were modeled using (3 × 3), (2 × 3), (3 × 3) and (3 × 4) atom containing surface unit cells with four, six, four and four layers, respectively. (3 × 3 × 1) Monkhorst-Pack mesh was used for (100), (110) and (111) surfaces, while (3 × 2 × 1) was used for (211) surface. In all calculations, adsorbates and topmost two layers were relaxed, while the other atoms were fixed to the optimized bulk positions.

To estimate a stability of Te dopant, we calculated a formation energy of Te doped surface, where Te is doped in the first and second layer of Pd slabs. The formation energy was estimated as $E_f = E_{Te/Pd} - n_{Pd}E_{Pd,\ bulk} - n_{Te}E_{Te,\ bulk}$, where $E_{Te/Pd}$, $E_{Pd,\ bulk}$, and $E_{Te,\ bulk}$ denote electronic energies of Te doped slab, bulk energies of Pd and Te per atom, respectivley. The relative formation energies (ΔE_f) were then plotted relative to E_f of (111) surface, where the formation of Te is most favorable (Figure S10).

To comparatively investigate the catalytic activity and selectivity of CO_2 electrocatalysis for Pd and Te/Pd catalysts, we converted the calculated electronic energies into free energies by adding free energy corrections using Atomic Simulation Environment (ASE) code.[9] For adsorbates, these corrections include zero point energies, enthalpy, and entropy corrections, which are calculated from a harmonic oscillator approximation with a finite displacement of ±0.01 Å in x-, y- and z-directions.

All the values are summarized in Table S1. For molecules, correction values were taken from Ref.[10] We further added +0.45 eV for CO_2 molecule to correct the inaccuracy of RPBE with respect to the experimental reaction energies.[10] To include the effect of water, we added an approximate solvation corrections, where *COOH and *CO were stabilized by 0.25 and 0.1 eV, respectively.[10] We used the computational hydrogen electrode (CHE) model[11] to estimate the chemical potential of proton and electron pair since CO_2 reduction reaction consists of two sequential protonation steps. In the CHE method, the chemical potential of proton and electron ($\mu(H^+ + e^-)$) is estimated from the half of chemical potential of H_2 gas ($0.5\mu(H_2)$) and the relation between chemical and electrical potential ($\Delta G = -eU$), i.e., $\mu(H^+ + e^-) = 0.5\mu(H_2) - eU$.

Table S1. The calculated free energy corrections for adsorbates. All values are given in eV.

	ZPE	$\int C_p dT$	−TS
*CO	0.187	0.031	-0.049
*COOH	0.599	0.088	-0.185
*H	0.161	0.005	-0.006

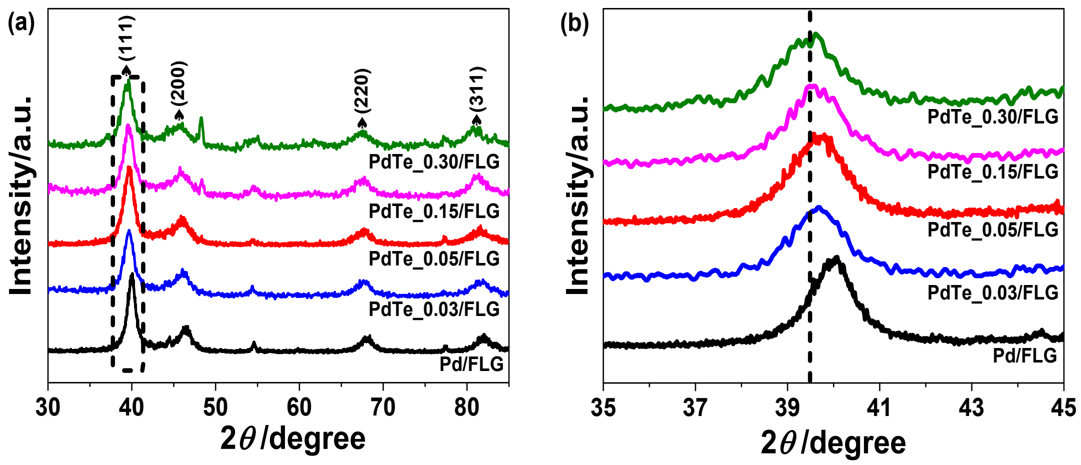

Fig. S1 XRD patterns of 30 wt% Pd/FLG, 30 wt% PdTe_0.03/FLG, 30 wt% PdTe_0.05/FLG, 30 wt% PdTe_0.15/FLG and 30 wt% PdTe_0.30/FLG over the range of (a) 30-85° and (b) 35-45°.

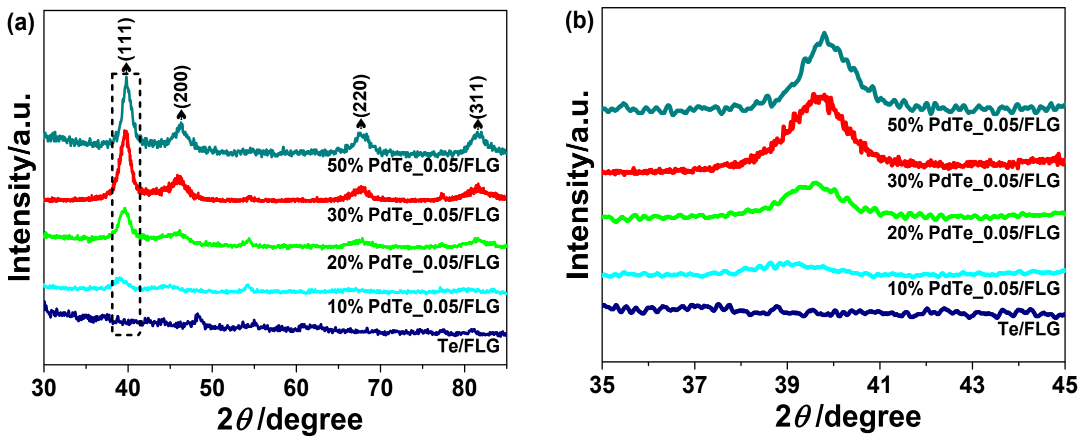

Fig. S2 XRD patterns of 30 wt% Te/FLG, 10 wt% PdTe_0.05/FLG, 20 wt% PdTe_0.05/FLG, 30 wt% PdTe_0.05/FLG, 50 wt% PdTe_0.05/FLG over the range of (a) 30-85°and (b) 35-45°.

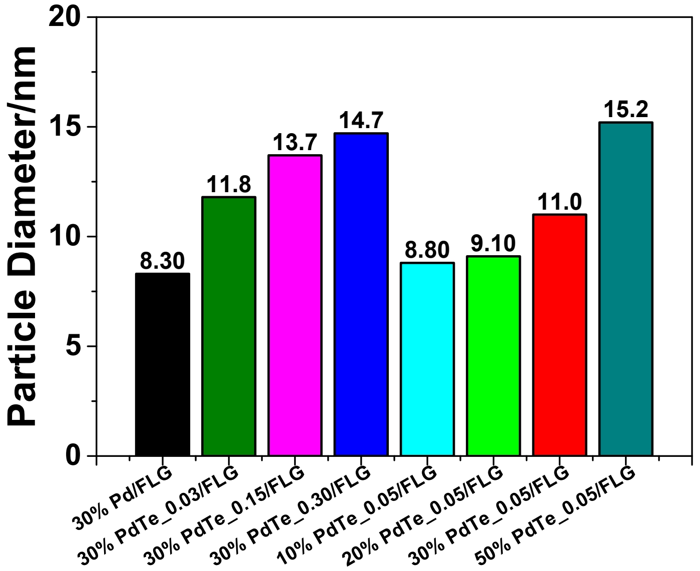

Fig. S3 The particle sizes of Pd and PdTe in 30 wt% Pd/FLG, 30 wt% PdTe_0.03/FLG, 30 wt% PdTe_0.15/FLG, 30 wt% PdTe_0.30/FLG, 10 wt% PdTe_0.05/FLG, 20 wt% PdTe_0.05/FLG, 30 wt% PdTe_0.05/FLG, and 50 wt% PdTe_0.05/FLG were estimated from the Pd (111) reflection utilizing Scherrer's equation relating the coherently scattering domains with Bragg peak widths: $L = k\lambda/B\cos(\theta)$, where k = 0.89 for spherical particles and B is the full angular width at half-maximum of the peak in radians.

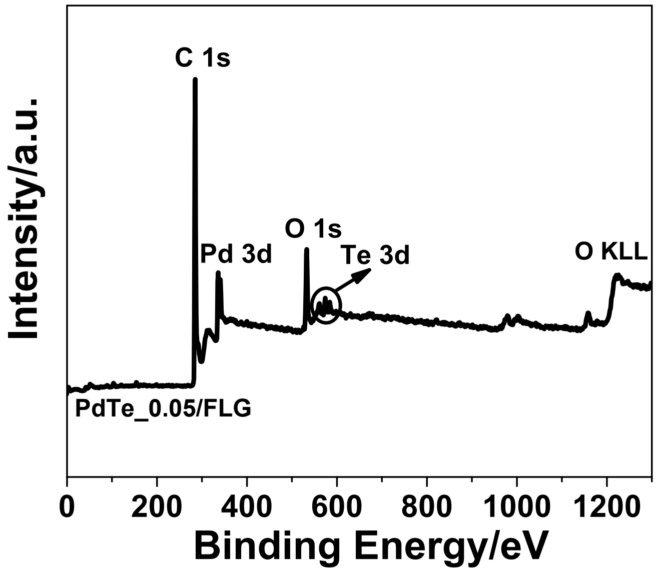

Fig. S4 The wide-survey XPS spectrum of 30 wt% PdTe_0.05/FLG.

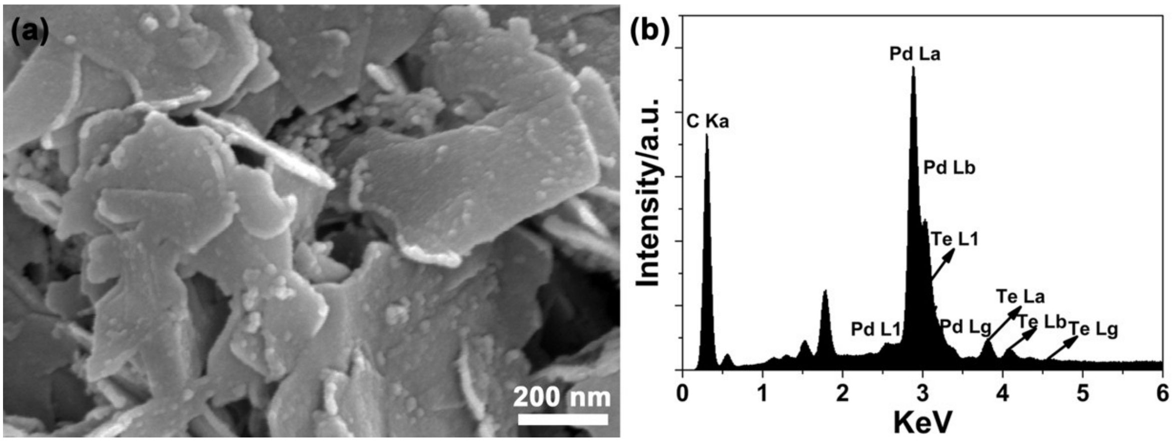

Fig. S5 (a) High-magnification SEM image and (b) EDX pattern of 30 wt% PdTe_0.05/FLG.

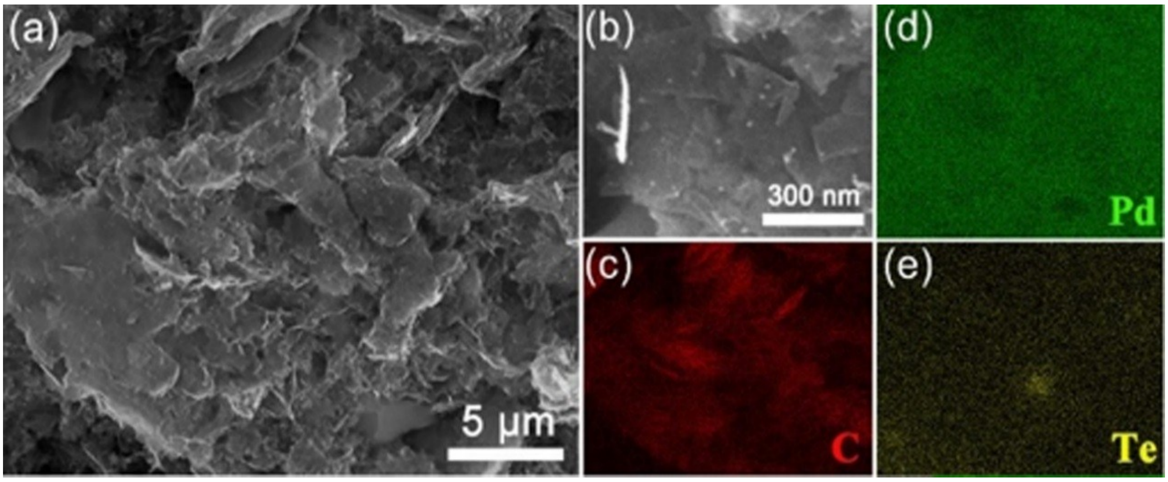

Fig. S6 (a) Low-, and (b) high-magnification SEM images of 30 wt% PdTe_0.05/FLG. (c)–(e) EDX mapping images of Pd, C, and Te, respectively, taken from the region shown in (a).

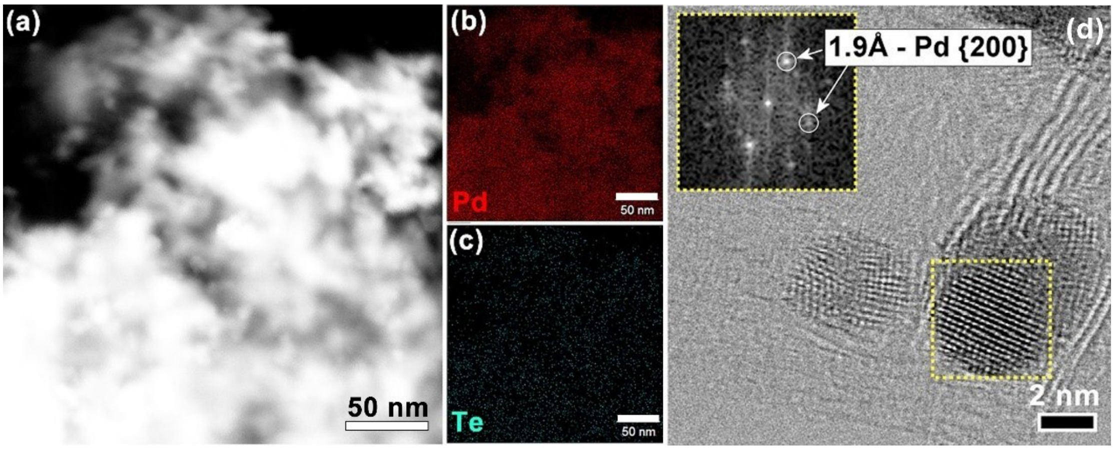

Fig. S7 (a) STEM image. (b) and (c) EDX mapping images of Pd and Te, respectively, taken from the region shown in (a). (d) High-magnification bright field STEM image of 30 wt% PdTe_0.05/FLG. Inset: FFT of the region marked in the yellow square.

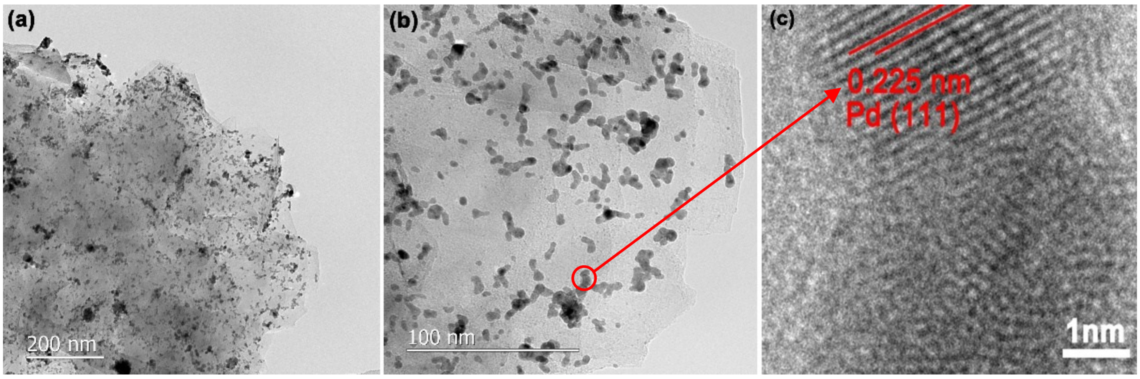

Fig. S8 (a) Low- and (b) high-magnification TEM images of 30 wt% Pd/FLG. (c) High-resolution TEM image of the particles marked in red in image b. Image c illustrates the high crystallinity of Pd NPs with an interplanar spacing of 0.225 nm, corresponding to the {111} facets of Pd.

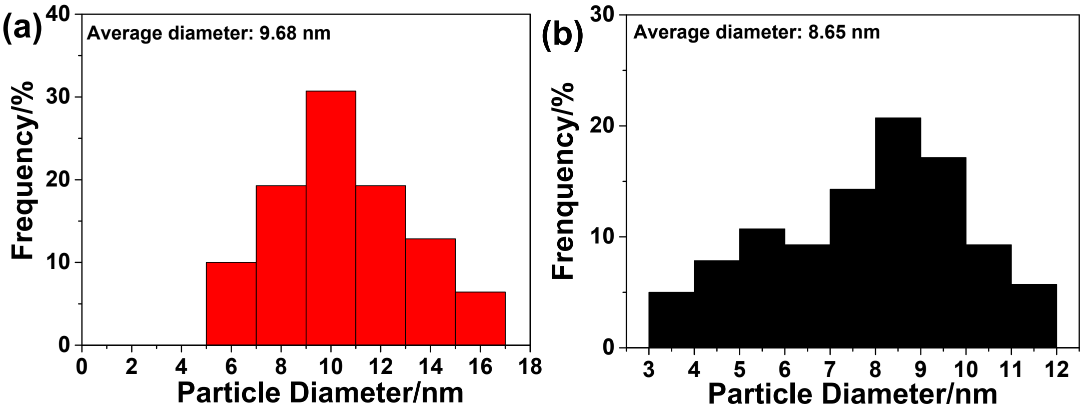

Fig. S9 The particle size distributions of (a) PdTe NPs in 30 wt% PdTe_0.05/FLG, and (b) Pd NPs in 30 wt% Pd/FLG extracted by counting 140 different individual particles on the basis of STEM (Fig. 2a for PdTe) and TEM (Fig. S8b for Pd) observations, respectively.

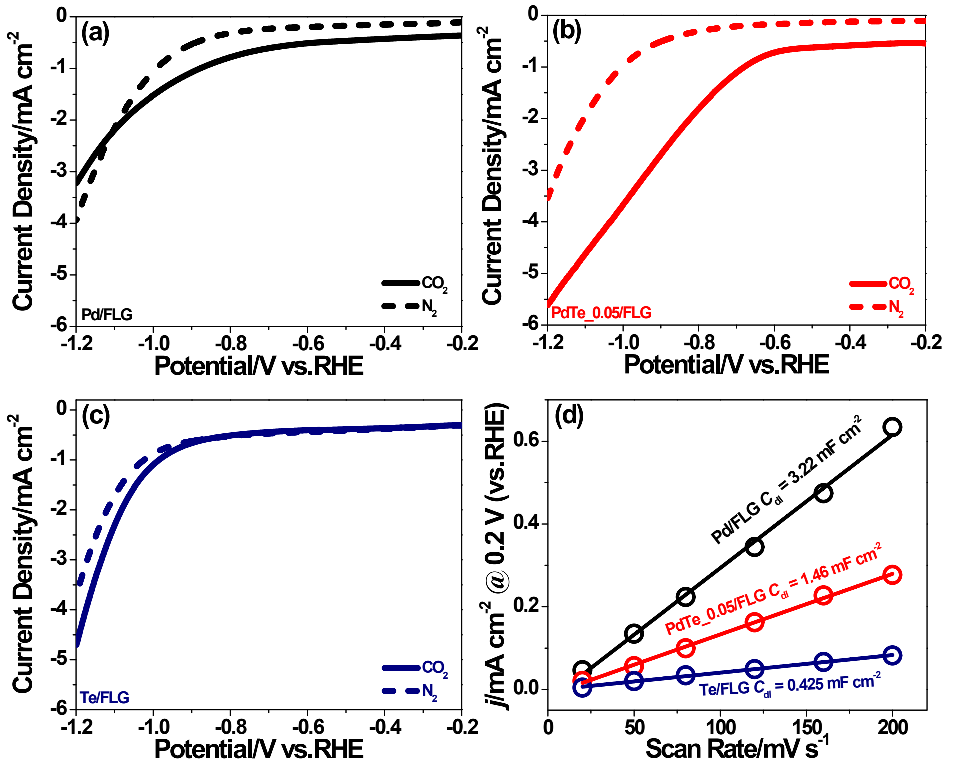

Fig. S10 Current–voltage curves on (a) 30 wt% Pd/FLG, (b) 30 wt% PdTe_0.05/FLG and (c) 30 wt% Te/FLG electrodes obtained from the LSV scans in CO_2- and N_2-saturated 0.1 M aqueous $KHCO_3$. Scan rate: 5 mV s^{-1}. (d) Current density of CV experiments at potential + 0.2 V (vs. RHE) as a function of scan rate. The slope of this line shows double-layer capacitor for each catalyst.

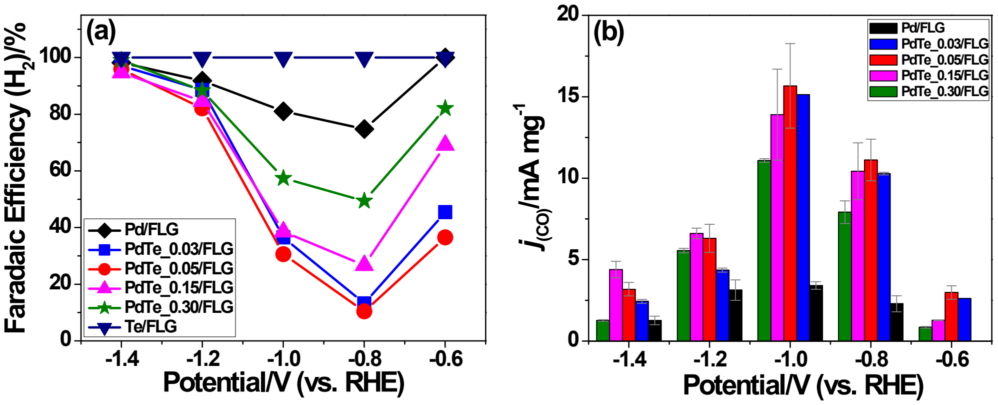

Fig. S11 (a) Faradaic efficiency for H_2 production of 30 wt% Pd/FLG, 30 wt% Te/FLG, and 30 wt% PdTe/FLG catalysts, and (b) mass activities of 30 wt% Pd/FLG, and 30 wt% PdTe/FLG with different Te doping contents at various applied potentials in 0.1 M aqueous $KHCO_3$.

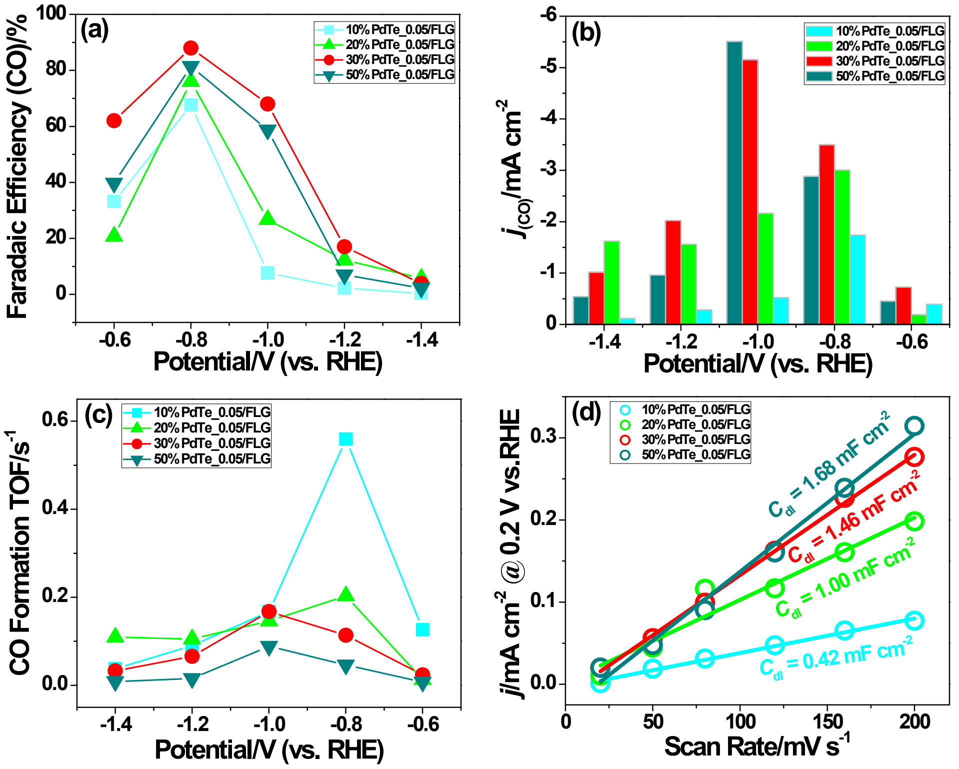

Fig. S12 (a) Faradaic efficiency, (b) partial current density, and (c) TOF of CO formation on different PdTe_0.05 mass loadings at various applied potentials in 0.1 M $KHCO_3$ electrolyte. (d) Current density of CV experiments at potential + 0.2 V (vs.

RHE) as a function of scan rate. The slope of this line shows double layer capacitor for each catalyst.

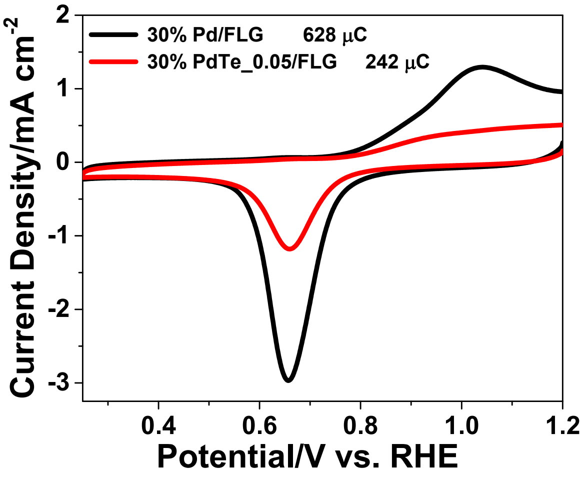

Fig. S13 Representative CVs of 30 wt% Pd/FLG and 30 wt% PdTe_0.05/FLG electrodes in a deaerated 0.1 M $HClO_4$ solution with a scanning rate of 50 mV s^{-1} with upper potential limits at +1.2 V (vs. RHE). The values of integrated charge for 30 wt% Pd/FLG and 30 wt% PdTe_0.05/FLG were thus derived to be about 628 and 242 μC, respectively.

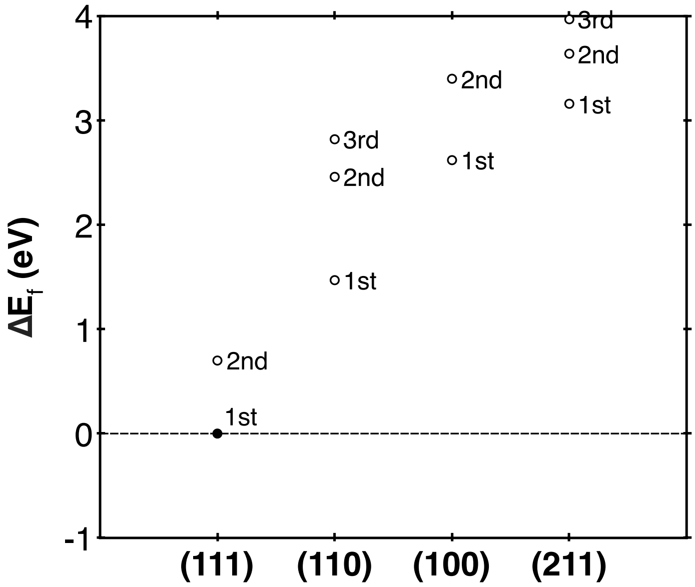

Fig. S14 The relative formation energies (ΔE_f) of Te doping on various layers of various Pd facets. All the energies were relative to the first layer Te doping on (111) surface, which is the most favorable doping site.

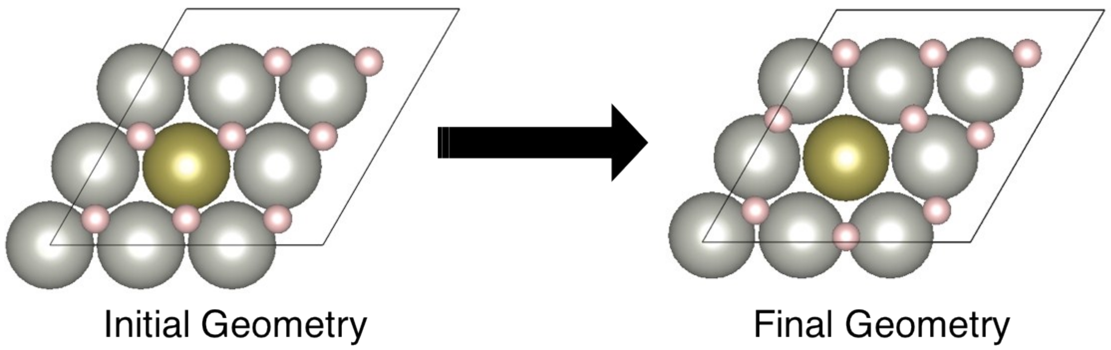

Fig. S15 Initial and final geometries of 1 ML *H-Te/Pd (111). Grey, gold and white balls indicate Pd, Te and H atom, respectively. As Te repelled nearby three H atoms during the optimization, we removed those H atoms for further calculations, denoted as 2/3 ML *H-Te/Pd (111) in Figure 4d.

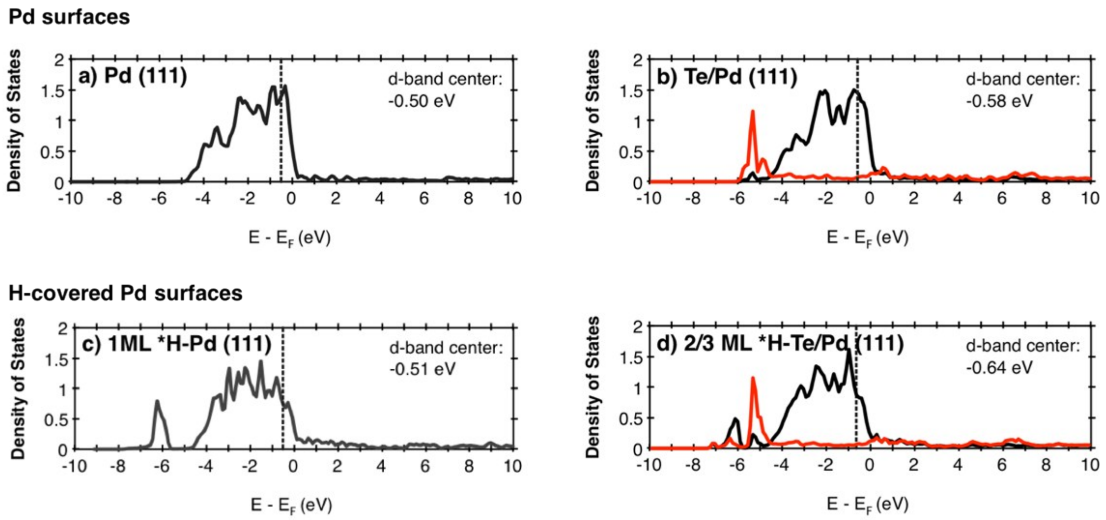

Fig. S16 The density of states analysis for Pd atom with (right) and without (left) Te dopant of Pd surface (upper) and H-covered Pd surface (lower). Black and red lines indicate 4*d* orbital of Pd and 5*p* orbital of Te. The d-band center of Pd atom is also shown.

References

1. L. Xiao, L. Zhuang, Y. Liu, J. T. Lu and H. D. Abruna, *J. Am. Chem. Soc.*, 2009, **131**, 602-608.
2. M. Hara, U. Linke and T. Wandlowski, *Electrochim. Acta*, 2007, **52**, 5733-5748.
3. M. Asadi, K. Kim, C. Liu, A. V. Addepalli, P. Abbasi, P. Yasaei, P. Phillips, A. Behranginia, J. M. Cerrato, R. Haasch, P. Zapol, B. Kumar, R. F. Klie, J. Abiade, L. A. Curtiss and A. Salehi-Khojin, *Science*, 2016, **353**, 467-470.
4. G. Kresse, *Phys. Rev. B*, 1996, **54**, 169.
5. J. P. Perdew, K. Burke and M. Ernzerhof, *Physical review letters*, 1996, **77**, 3865.
6. B. Hammer, L. B. Hansen and J. K. Nørskov, *Phys. Rev. B*, 1999, **59**, 7413.
7. P. E. Blöchl, *Phys. Rev. B*, 1994, **50**, 17953.
8. G. Kresse and D. Joubert, *Phys. Rev. B*, 1999, **59**, 1758.
9. S. R. Bahn and K. W. Jacobsen, *Comput. Sci. Eng.*, 2002, **4**, 56-66.
10. A. A. Peterson, F. Abild-Pedersen, F. Studt, J. Rossmeisl and J. K. Norskov, *Energ. Environ. Sci.*, 2010, **3**, 1311-1315.
11. J. K. Nørskov, J. Rossmeisl, A. Logadottir, L. Lindqvist, J. R. Kitchin, T. Bligaard and H. Jonsson, *The J. Phys. Chem. B*, 2004, **108**, 17886-17892.
